# Data-driven classification of primary Sjögren’s syndrome: From cluster analysis to clinical immune phenotypes and predictive biomarkers

**DOI:** 10.1016/j.jtauto.2025.100338

**Published:** 2025-12-03

**Authors:** Jianbin Li, Suiran Li, Wei Liu

**Affiliations:** a Department of Rheumatism and Immunity, First Teaching Hospital of Tianjin University of Traditional Chinese Medicine, Tianjin, China; b National Clinical Research Center for Chinese Medicine Acupuncture and Moxibustion, Tianjin, China

**Keywords:** Sjögren’s syndrome, Clustering analysis, Network analysis, Clinical phenotypes, Biomarkers, Systemic involvement

## Abstract

**Background:**

Primary Sjögren’s Syndrome (pSS) exhibits significant clinical heterogeneity, and traditional organ-based classification systems fail to capture the underlying disease mechanisms. This study aims to identify distinct clinical immune phenotypes of pSS through a data-driven approach and explore their predictive biomarkers.

**Method:**

This cross-sectional study included 1087 patients who met the 2016 ACR/EULAR classification criteria for primary Sjögren’s syndrome between 2014 and 2024. Unsupervised K-means clustering analysis was applied to 10 organ involvement variables to identify natural patient subgroups. Network analysis was used to explore the associations between organ involvement and laboratory biomarkers. Multivariable logistic regression was employed to identify independent predictors of subgroup assignment, and restricted cubic spline analysis was conducted to assess the nonlinear relationships between key biomarkers and subtype risk.

**Results:**

Clustering analysis identified two distinct phenotypes: Phenotype 1 (multi-system inflammatory subtype, n = 594) was characterized by widespread musculoskeletal involvement (100 %) and significantly elevated inflammatory markers (RF: 246.41 ± 1177.49 vs 32.75 ± 126.74 IU/mL, P < 0.001); Phenotype 2 (glandular-limited high immunoglobulin subtype, n = 493) was primarily characterized by glandular involvement (40.7 %), higher IgG levels, and less systemic involvement. Network analysis revealed a strong correlation between RF and musculoskeletal involvement (r = 0.32, P < 0.001). Independent predictors of Phenotype 1 included male gender (OR 2.559, 95 % CI 1.109–6.090), elevated potassium (OR 1.607, 95 % CI 1.061–2.433), and elevated RF levels (OR 1.004, 95 % CI 1.002–1.005). A composite clinical prediction score incorporating these biomarkers achieved an AUC of 0.717 (95 % CI: 0.684–0.751) for phenotype discrimination. Nonlinear analysis showed complex U-shaped and inverted U-shaped relationships between key biomarkers and phenotype risk.

**Conclusion:**

pSS consists of distinct clinical phenotypes with varying pathophysiological characteristics. The data-driven classification system complements traditional severity grading and provides new insights into precision medicine approaches. RF is a key biomarker linking musculoskeletal manifestations with the severity of systemic inflammation and may serve as an important indicator for precise subtyping and targeted therapy.

## Highlights

• Data-driven clustering reveals two distinct clinical phenotypes in primary Sjögren’s Syndrome.
• Rheumatoid factor links musculoskeletal involvement with systemic inflammation severity.
• A composite score using routine biomarkers achieves acceptable phenotype discrimination (AUC = 0.717).
• Nonlinear associations exist between laboratory parameters and disease phenotypes.

## 1 Introduction

Primary Sjögren’s Syndrome (pSS) is a chronic, systemic autoimmune disorder characterized by lymphocytic infiltration of the exocrine glands, primarily affecting the salivary and lacrimal glands, leading to dry eye and dry mouth symptoms [1]. With a global prevalence of 0.05–1 % and a notable female predominance (9:1 ratio), pSS is a common autoimmune disease that not only manifests as localized glandular damage but also affects multiple organ systems, including the joints, lungs, kidneys, nervous system, and vasculature [2]. The clinical manifestations of pSS exhibit significant heterogeneity, ranging from mild dryness symptoms to life-threatening complications such as lymphoma. This remarkable phenotypic variability poses significant challenges for accurate diagnosis, individualized treatment, and prognosis assessment.

Currently, traditional assessments of the severity of primary Sjögren’s syndrome (pSS) primarily rely on the number of affected organs. However, this approach has significant limitations. For example, it fails to distinguish between involvement of a single life-threatening organ (such as severe pulmonary interstitial disease) and multiple organs with mild symptoms, potentially leading to inaccurate assessments of disease severity. This “one-size-fits-all” approach does not adequately capture the underlying pathophysiological mechanisms driving the different clinical manifestations. Although scoring systems such as ESSDAI, used clinically, provide standardized tools for assessing disease activity, they do not account for different biological processes that may underlie seemingly similar clinical presentations. Recent studies have shown that pSS patients exhibit distinct clinical subtypes and immunological features, suggesting the presence of multiple disease-driving mechanisms [[3], [4]]. However, there is currently a lack of studies based on large cohorts that use data-driven methods to systematically identify these natural disease clusters.

The advancement of machine learning and network medicine techniques has provided unprecedented opportunities to dissect disease heterogeneity and identify clinically relevant subtypes. Unsupervised clustering algorithms, which identify patient subgroups based on similarity patterns without prior assumptions, offer the potential to uncover novel disease subtypes with differing therapeutic responses and prognoses. Such methods have been successfully applied in autoimmune diseases such as rheumatoid arthritis and systemic lupus erythematosus, but their application in pSS remains limited [5,6]. More challenging is the fact that although anti-Ro/SSA and anti-La/SSB are hallmark serological antibodies for pSS, they can also be found in other autoimmune diseases, such as systemic lupus erythematosus, and even in some healthy individuals. This further complicates the diagnosis and precise classification of the disease.

In light of this, the objectives of this study are: (1) to identify distinct clinical phenotypes in pSS through unsupervised clustering of organ involvement patterns in a large cohort; (2) to construct a network linking organ involvement and laboratory biomarkers; (3) to validate data-driven subtypes against traditional severity classifications; (4) to develop predictive models for subtype classification; and (5) to explore the nonlinear relationships between biomarkers and phenotype risk to enhance phenotype characterization. Through this multi-layered analytical approach, we aim to gain deeper insights into the heterogeneity of pSS and lay the foundation for future precision medicine strategies.

## 2 Methods

### 2.1 Study design and participants

The data for this single-center, cross-sectional study were derived from the electronic medical record system of the First Affiliated Hospital of Tianjin University of Traditional Chinese Medicine. We retrospectively included 1087 patients who met the 2016 American College of Rheumatology/European League Against Rheumatism (ACR/EULAR) classification criteria for primary Sjögren’s syndrome between January 2014 and December 2024 [7]. To ensure cohort consistency, we retrospectively applied the 2016 classification criteria for patients admitted between 2014 and 2015. The study collected demographic data, clinical manifestations, laboratory parameters, and treatment information at baseline. The study protocol was approved by the Ethics Committee of the First Affiliated Hospital of Tianjin University of Traditional Chinese Medicine (approval number: TYLL2018[K]026). Given its retrospective design, the Ethics Committee waived the requirement for informed consent from patients.

### 2.2 Clinical assessment and disease severity classification

We assessed the systemic involvement of patients by reviewing their electronic medical records. Systemic involvement was defined as the presence of clear clinical diagnoses of organ dysfunction or pathological changes, documented by a rheumatologist, regardless of whether the condition was currently in an active phase. Although we referred to the 12 organ domains outlined in the EULAR Sjögren’s Syndrome Disease Activity Index (ESSDAI) as an assessment framework [8], our criteria focused on the presence of organ involvement rather than the disease activity level measured by ESSDAI. The evaluation covered ten major organ systems: glands, lungs, nervous system, blood, liver, kidneys, musculoskeletal system, cardiovascular system, digestive system, and endocrine system. To ensure data accuracy, the systemic involvement of each patient was independently reviewed and determined by two experienced rheumatologists (with good inter-rater agreement, Cohen’s κ = 0.87), and any discrepancies were resolved by a third senior expert. Additionally, for comparison with traditional clinical practice, we initially grouped patients into three severity categories based on the number of affected systems: mild (Group 0, only glandular involvement), moderate (Group 1, 1–2 non-glandular systems involved), and severe (Group 2, ≥3 systems involved). This study will later explore the similarities and differences between this traditional grouping and the data-driven subtyping.

### 2.3 Laboratory testing

All patients underwent standardized comprehensive laboratory evaluations, including complete blood count, liver and kidney function tests, lipid profile, electrolytes, blood glucose, immunoglobulins (IgG, IgA, IgM), complement components (C3, C4), and autoantibodies (anti-Ro/SSA, anti-La/SSB) detected by linear immunoblotting. The kits used for these tests were provided by EUROIMMUN Medizinische Labordiagnostika AG (Lübeck, Germany). In addition, inflammation markers (ESR, CRP) and rheumatoid factor (RF, measured by nephelometry, with a positive threshold >20 IU/mL) were also assessed. All tests were conducted at the central laboratory of the First Affiliated Hospital of Tianjin University of Traditional Chinese Medicine, following international standardization procedures and the instrument manufacturers’ protocols, with strict quality control measures in place. All laboratory data were checked for outliers, and any values exceeding the physiologically plausible range were considered as missing data.

### 2.4 Statistical analysis

#### 2.4.1 Clustering analysis

Unsupervised k-means clustering based on organ involvement patterns was performed to identify patient subgroups [9]. Prior to clustering, binary system involvement variables (coded as 0 = no involvement, 1 = involvement) were standardized using Z-scores to ensure comparability across variables. Missing data (<5 %) were handled using multiple imputation, generating five complete datasets for analysis. The optimal number of clusters was determined using several methods to enhance the robustness of the results: (1) silhouette coefficient method, evaluating clustering solutions from k = 2 to k = 6; (2) gap statistic; (3) elbow method; (4) clinical interpretability. The comprehensive evaluation indicated that k = 2 was the optimal choice (mean silhouette coefficient = 0.68, higher than all other k values). K-means clustering was performed using Euclidean distance and implemented with the Lloyd algorithm, with 100 repetitions of different random starting points (nstart = 25) to ensure a global optimal solution and stable clustering results. Bootstrap sampling (1000 repetitions) was used to assess the stability of the clustering solution, yielding a high Jaccard coefficient (0.78), indicating stable clustering. Principal component analysis (PCA) was applied for dimensionality reduction and clustering visualization, with the first two principal components explaining 63.7 % of the total variance.

### 2.5 Network analysis

Network analysis was performed using the “igraph” and “ggraph” packages in R (version 4.1.0) to explore associations between organ involvement and laboratory biomarkers. Spearman’s rank correlation coefficients were used to assess relationships between variables, adapting for non-normal data distribution. The network construction involved the following steps: (1) calculating the Spearman correlation matrix and corresponding p-value matrix for all variable pairs; (2) retaining only statistically significant correlations (P < 0.05, without multiple comparison corrections); (3) constructing a bipartite graph where nodes represent system involvement variables (n = 10) and laboratory parameters (n = 10), and edges represent significant correlations; (4) edge thickness and color intensity were based on the absolute value of the correlation coefficient. To enhance visualization, the Fruchterman-Reingold algorithm was applied for network layout optimization. Nodes in the network were color-coded by type (system involvement variables in blue, laboratory markers in orange).

### 2.6 Nonlinear relationship analysis

To explore the complex nonlinear relationships between laboratory parameters and subtype classification, a restricted cubic spline (RCS) model was used, with knots at the 5th, 35th, 65th, and 95th percentiles. This approach allows flexible modeling of nonlinear associations between continuous variables and outcomes while avoiding overfitting. For each laboratory parameter, two logistic regression models were fitted: one including spline terms and the other including only linear terms. A likelihood ratio test was used to compare these models (P < 0.05 indicating significant nonlinearity). The degrees of freedom in the spline model were optimized based on the Akaike information criterion (AIC), with the final model selection considering model fit and clinical interpretability. RCS analysis was implemented using the “rms” package in R, and risk relationship curves and their 95 % confidence intervals were visualized using the “ggplot2” package.

### 2.7 Predictor analysis

Logistic regression was used to identify predictors for subtype classification. All demographic, clinical, and laboratory variables were evaluated for their association with subtype classification in univariate analysis, with variables showing P < 0.2 included in the multivariate model. In the multivariate model, Phenotype 1 (multi-system inflammatory subtype) was defined as 1, and Phenotype 2 (glandular-limited high immunoglobulin subtype) as 0. Stepwise backward elimination (based on AIC) was used to optimize the final model. Odds ratios (OR) with 95 % confidence intervals (CI) were calculated, and the goodness-of-fit of the model was evaluated using the Hosmer-Lemeshow test (P = 0.78, indicating good model fit). Multicollinearity was assessed using the variance inflation factor (VIF), and variables with VIF >5 were excluded from the final model. The model’s discriminatory ability was assessed using the area under the ROC curve (AUC = 0.74, 95 % CI 0.71–0.77). All statistical analyses were performed using R software (version 4.1.0) and SPSS (version 25.0), with a two-tailed P < 0.05 considered statistically significant.

### 2.8 Composite clinical prediction score development

To enhance the clinical applicability of our classification system, we developed a composite prediction score based on the independent predictors identified in the multivariate logistic regression analysis. The model incorporated eight routine laboratory and demographic variables: rheumatoid factor (RF), erythrocyte sedimentation rate (ESR), albumin, gender, potassium (K), glucose, gamma-glutamyl transferase (GGT), and immunoglobulin G (IgG). The discriminatory performance of the composite score was evaluated using receiver operating characteristic (ROC) curve analysis. The area under the ROC curve (AUC) with 95 % confidence interval was calculated using the DeLong method. The optimal cut-off value was determined using Youden’s index (sensitivity + specificity − 1), and the corresponding sensitivity and specificity were reported.

## 3 Results

### 3.1 Baseline characteristics and clinical manifestations of patients

Table 1 presents the demographic and clinical characteristics of 1087 patients with Sjögren’s syndrome, stratified by disease severity (mild, moderate, and severe). The mean age of all patients was 56.88 ± 12.15 years, with a predominance of females (93.6 %). No significant differences were observed in age, gender distribution, or BMI across the severity groups. Several laboratory parameters showed significant differences between severity groups. Liver function indicators, particularly ALT, were significantly lower in the moderate and severe groups compared to the mild group (P = 0.024). Total protein and albumin levels decreased with increasing disease severity (P = 0.029 and P = 0.001, respectively). Inflammatory markers were significantly associated with disease severity, with ESR and CRP levels gradually increasing from mild to severe groups (P = 0.003 and P = 0.015, respectively). Notably, rheumatoid factor (RF) significantly increased with disease severity, rising from 25.82 ± 52.74 IU/mL in the mild group to 275.36 ± 1707.09 IU/mL in the severe group (P = 0.041). The organ involvement patterns differed significantly between severity groups. The mild group was primarily characterized by glandular involvement, with minimal non-glandular manifestations. The moderate group showed increased involvement of the musculoskeletal system (393 cases), cardiovascular system (111 cases), and lungs (65 cases). The severe group (Group 2) exhibited the highest rates of involvement in the lungs, cardiovascular, digestive, and endocrine systems, consistent with the definition of increased disease severity. Regarding comorbidities, hypertension was the most common (171 cases), followed by diabetes (33 cases) and pulmonary interstitial fibrosis (62 cases). These comorbidities exhibited significant differences between severity groups (P < 0.01), with higher prevalence rates in the moderate and severe groups. Notably, lymphoma, though rare (7 cases), had a significantly higher prevalence in the severe group (P = 0.001).。

**Table 1:** Demographic characteristics and clinical manifestations of Sjögren’s syndrome patients.

Character	Total (n = 1087)	Mild Group (n = 200)	Moderate Group (n = 656)	Severe Group (n = 231)	P-value
Age (years)	58.25 ± 12.16	56.88 ± 12.15	58.61 ± 12.27	58.41 ± 11.81	0.205
Female, n (%)	1017(93.56)	188(94.00)	612(93.29)	217(93.94)	0.906
BMI (kg/m^2^)	23.77 ± 4.76	23.62 ± 4.27	23.73 ± 4.73	24.05 ± 5.29	0.661
Alanine Aminotransferase (U/L)	25.19 ± 40.09	31.51 ± 63.35	24.76 ± 34.26	20.99 ± 26.97	0.024
Aspartate Aminotransferase (U/L)	28.36 ± 58.99	34.26 ± 76.91	28.85 ± 62.43	21.94 ± 12.90	0.095
Alkaline Phosphatase (U/L)	79.05 ± 43.43	78.51 ± 50.53	79.44 ± 43.18	78.41 ± 37.32	0.936
Gamma-Glutamyl Transferase (U/L)	36.21 ± 65.23	37.19 ± 70.85	36.98 ± 64.94	33.20 ± 61.09	0.734
Total Protein (g/L)	68.19 ± 8.52	69.09 ± 8.94	68.34 ± 8.69	66.97 ± 7.51	0.029
Albumin (g/L)	35.79 ± 4.65	36.87 ± 4.41	35.64 ± 4.74	35.29 ± 4.46	0.001
Creatinine (μmol/L)	61.97 ± 25.13	61.54 ± 19.29	62.82 ± 28.28	59.91 ± 19.49	0.316
Cholesterol (mmol/L)	4.39 ± 1.13	4.46 ± 1.23	4.37 ± 1.10	4.38 ± 1.14	0.675
Triglyceride (mmol/L)	1.35 ± 0.81	1.39 ± 0.91	1.35 ± 0.80	1.34 ± 0.77	0.816
High-Density Lipoprotein (mmol/L)	1.12 ± 0.34	1.17 ± 0.37	1.12 ± 0.34	1.10 ± 0.34	0.125
Low-Density Lipoprotein (mmol/L)	2.62 ± 0.86	2.62 ± 0.91	2.62 ± 0.86	2.63 ± 0.83	0.98
Lactate Dehydrogenase (U/L)	190.56 ± 85.10	188.93 ± 74.45	192.20 ± 94.21	187.34 ± 64.11	0.735
Hydroxybutyrate Dehydrogenase (U/L)	143.85 ± 66.04	142.53 ± 55.45	145.53 ± 73.55	140.26 ± 50.15	0.568
Potassium (mmol/L)	3.87 ± 0.42	3.83 ± 0.45	3.87 ± 0.42	3.89 ± 0.39	0.32
Sodium (mmol/L)	140.32 ± 2.87	140.32 ± 2.74	140.34 ± 2.97	140.30 ± 2.71	0.983
Chloride (mmol/L)	107.46 ± 3.51	107.64 ± 3.70	107.43 ± 3.58	107.41 ± 3.13	0.742
Calcium (mmol/L)	2.18 ± 0.12	2.19 ± 0.13	2.18 ± 0.13	2.17 ± 0.12	0.19
Magnesium (mmol/L)	0.85 ± 0.09	0.86 ± 0.09	0.86 ± 0.09	0.85 ± 0.09	0.207
Phosphate (mg/dL)	78.93 ± 43.55	78.12 ± 50.83	79.44 ± 43.18	78.21 ± 37.61	0.897
Blood Glucose (mmol/L)	5.04 ± 1.34	4.90 ± 0.97	5.10 ± 1.47	4.99 ± 1.23	0.156
Globulin (g/L)	5.15 ± 10.53	6.85 ± 12.10	4.89 ± 10.33	4.42 ± 9.50	0.037
Immunoglobulin G (g/L)	12.69 ± 18.55	12.34 ± 3.92	12.93 ± 23.69	12.35 ± 3.54	0.878
Immunoglobulin A (g/L)	2.39 ± 1.04	2.38 ± 1.12	2.35 ± 0.99	2.52 ± 1.10	0.112
Immunoglobulin M (g/L)	1.26 ± 0.65	1.28 ± 0.67	1.23 ± 0.59	1.35 ± 0.80	0.07
Complement C3 (g/L)	1.09 ± 0.36	1.07 ± 0.36	1.09 ± 0.36	1.10 ± 0.34	0.579
Complement C4 (g/L)	0.22 ± 0.10	0.22 ± 0.09	0.22 ± 0.11	0.23 ± 0.09	0.95
Erythrocyte Sedimentation Rate (mm/hr)	47.90 ± 31.53	40.44 ± 30.24	49.29 ± 31.94	50.36 ± 30.69	0.003
Anti-Streptolysin O (IU/mL)	89.89 ± 79.67	88.96 ± 55.11	88.07 ± 81.34	95.99 ± 91.98	0.506
C-Reactive Protein (mg/L)	14.56 ± 25.57	9.56 ± 18.17	15.10 ± 25.96	17.33 ± 29.22	0.015
Rheumatoid Factor (IU/mL)	157.40 ± 908.86	25.82 ± 52.74	156.12 ± 590.06	275.36 ± 1707.09	0.041
SSA positive, n (%)	671 (61.73 %)	126 (63.00 %)	402 (61.28 %)	143 (61.90 %)	0.907
SSB positive, n (%)	211 (19.41 %)	39 (19.50 %)	126 (19.21 %)	46 (19.91 %)	0.973
No Extra-glandular Involvement, n (%)	202 (18.58 %)	200 (100.00 %)	2 (0.30 %)	0 (0.00 %)	
Glandular involvement, n (%)	136 (12.51 %)	0 (0.00 %)	65 (9.91 %)	71 (30.74 %)	0.001
Pulmonary system involvement, n (%)	14 (1.29 %)	0 (0.00 %)	12 (1.83 %)	2 (0.87 %)	0.001
Nervous system involvement, n (%)	22 (2.02 %)	0 (0.00 %)	10 (1.52 %)	12 (5.19 %)	0.108
Hematologic system involvement, n (%)	7 (0.64 %)	0 (0.00 %)	5 (0.76 %)	2 (0.87 %)	0.001
Hepatic system involvement, n (%)	1 (0.09 %)	0 (0.00 %)	1 (0.15 %)	0 (0.00 %)	0.445
Renal system involvement, n (%)	594 (54.65 %)	0 (0.00 %)	393 (59.91 %)	201 (87.01 %)	0.720
Musculoskeletal system involvement, n (%)	193 (17.76 %)	0 (0.00 %)	111 (16.92 %)	82 (35.50 %)	0.001
Cardiovascular system involvement, n (%)	83 (7.64 %)	0 (0.00 %)	29 (4.42 %)	54 (23.38 %)	0.001
Digestive system involvement, n (%)	52 (4.78 %)	0 (0.00 %)	30 (4.57 %)	22 (9.52 %)	0.001
Endocrine system involvement, n (%)	171 (15.73 %)	0 (0.00 %)	99 (15.09 %)	72 (31.17 %)	0.001
Main Comorbidities, n (%)	33 (3.04 %)	0 (0.00 %)	21 (3.20 %)	12 (5.19 %)	
Hypertension, n (%)	12 (1.10 %)	0 (0.00 %)	6 (0.91 %)	6 (2.60 %)	0.001
Diabetes, n (%)	62 (5.70 %)	0 (0.00 %)	22 (3.35 %)	40 (17.32 %)	0.007
Hashimoto’s thyroiditis, n (%)	4 (0.37 %)	0 (0.00 %)	2 (0.30 %)	2 (0.87 %)	0.028
Interstitial fibrosis of lung, n (%)	7 (0.64 %)	0 (0.00 %)	1 (0.15 %)	6 (2.60 %)	0.001
Immune thrombocytopenia, n (%)	14 (1.29 %)	0 (0.00 %)	8 (1.22 %)	6 (2.60 %)	0.306
Lymphoma, n (%)	496 (45.63 %)	0 (0.00 %)	300 (45.73 %)	196 (84.85 %)	0.001
Malignant tumor, n (%)	250 (23.00 %)	27 (13.50 %)	160 (24.39 %)	63 (27.27 %)	0.056
Other autoimmune diseases, n (%)	55 (5.06 %)	12 (6.00 %)	35 (5.34 %)	8 (3.46 %)	0.001
Medication Use, n (%)	558 (51.33 %)	100 (50.00 %)	357 (54.42 %)	101 (43.72 %)	
Antihypertensive drugs, n (%)	85 (7.82 %)	15 (7.50 %)	51 (7.77 %)	19 (8.23 %)	0.001
Hypoglycemic agents, n (%)	460 (42.32 %)	81 (40.50 %)	283 (43.14 %)	96 (41.56 %)	0.428
Hormones/Steroids, n (%)	43 (3.96 %)	14 (7.00 %)	24 (3.66 %)	5 (2.16 %)	0.018
Tripterygium Wilfordii, n (%)	671 (61.73 %)	126 (63.00 %)	402 (61.28 %)	143 (61.90 %)	0.959
Hydroxychloroquine, n (%)	211 (19.41 %)	39 (19.50 %)	126 (19.21 %)	46 (19.91 %)	0.776
Total Glucosides of Paeony, n (%)	202 (18.58 %)	200 (100.00 %)	2 (0.30 %)	0 (0.00 %)	0.030

Note: Data are presented as mean ± standard deviation or n (%). A P-value <0.05 was considered statistically significant. Group comparisons were performed using one-way analysis of variance (ANOVA) for continuous variables and chi-square test for categorical variables.

### 3.2 Network of associations between organ involvement and laboratory markers

**Fig. 1:**
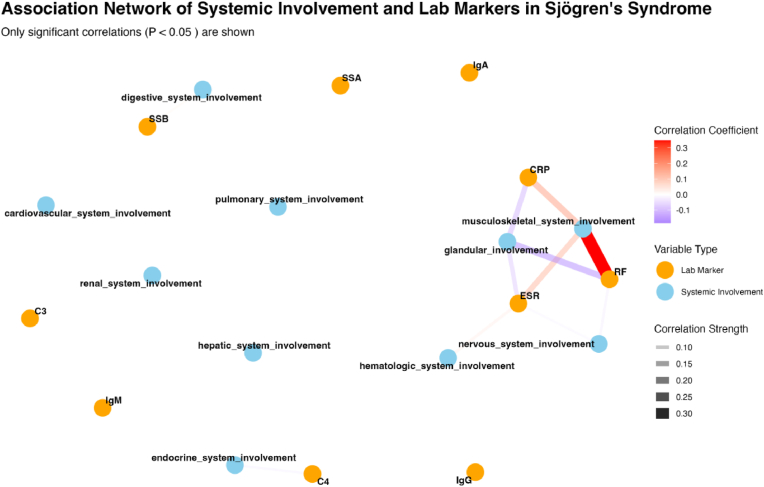
Association Network Between Organ Involvement and Laboratory Markers in Sjögren’s Syndrome. Note: Only statistically significant correlations (P < 0.05) are displayed. Line thickness and color intensity represent the strength of the correlation. Blue nodes represent system involvement variables, and orange nodes represent laboratory markers.

Fig. 1 illustrates the association network between the organ involvement patterns and laboratory markers in Sjögren’s Syndrome. This visualization highlights significant correlations (P < 0.05) between various clinical manifestations and laboratory parameters. A notable finding is the strong positive correlation between rheumatoid factor (RF) and musculoskeletal involvement (correlation coefficient r = 0.32, P < 0.001), represented by the thickest red connection line. This suggests that RF may be a key biomarker for joint manifestations in Sjögren’s syndrome. Additionally, inflammatory markers ESR and CRP show moderate correlations with musculoskeletal and glandular involvement (r = 0.18–0.24, P < 0.01), reflecting their role in tracking systemic inflammation. Several laboratory markers (IgG, IgM, C3, C4) appear as isolated nodes, indicating that they may have more generalized or complex relationships, rather than specific associations with particular organ systems. The network also reveals that certain organ systems, including the liver, kidneys, and blood, have fewer significant associations with the laboratory markers analyzed. This network analysis provides a novel visualization of the complex relationships between clinical manifestations and laboratory parameters in Sjögren’s syndrome, highlighting potential biomarkers for specific organ involvement patterns.

#### 3.2.1 Clustering analysis and patient subtypes

**Fig. 2:**
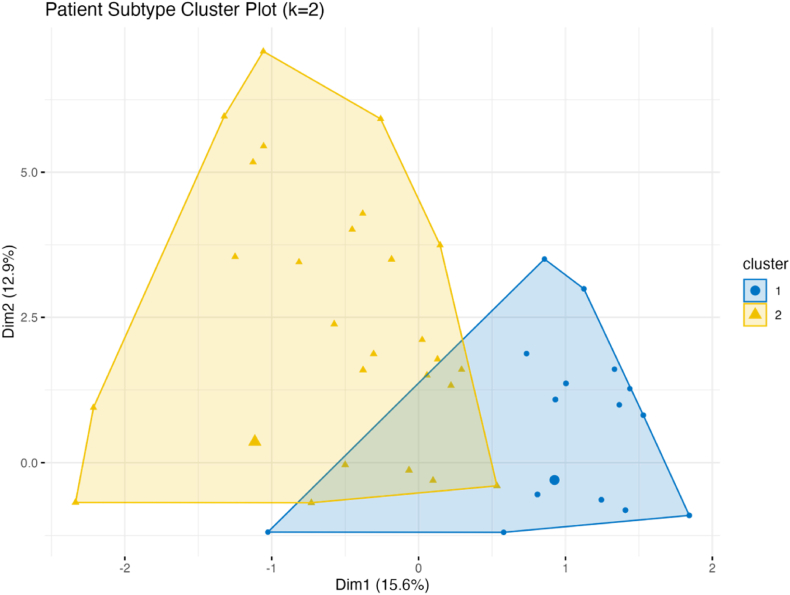
Clustering Plot of Sjögren’s Syndrome Patient Subtypes. Note: Unsupervised k-means clustering results based on 10 organ involvement variables. Blue points represent Subtype 1 (multi-system involvement - inflammation-dominant subtype), while yellow points represent Subtype 2 (glandular-limited - high immunoglobulin subtype). Dim1 and Dim2 represent the first two dimensions of principal component analysis.

**Fig. 3:**
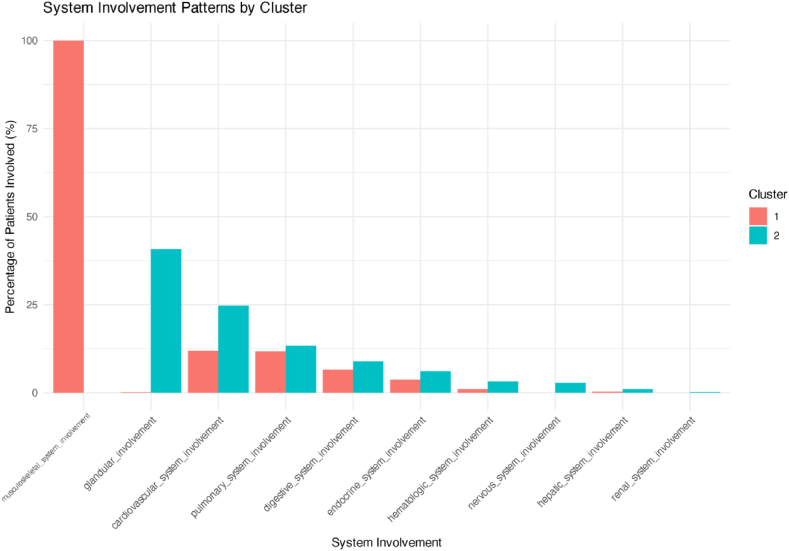
Comparison of Organ Involvement Patterns Between Sjögren’s Syndrome Subtypes. Note: The bar chart shows the percentage of patients with different organ involvement in each subtype. Red represents Subtype 1 (multi-system involvement - inflammation-dominant subtype), while blue represents Subtype 2 (glandular-limited - high immunoglobulin subtype).

Supplementary Fig. S1 presents the silhouette coefficient plot, identifying k = 2 as the optimal number of clusters, indicating that patients with Sjögren’s syndrome naturally divide into two distinct subgroups. Fig. 2 shows the distribution of patient subtypes based on unsupervised clustering, with the two clusters clearly separated in the two-dimensional space, with minimal overlap, reflecting the robustness of the subtype identification. The average silhouette coefficient of 0.68 suggests good clustering quality. Fig. 3 provides a detailed comparison of the organ involvement patterns between the two identified subtypes. Subtype 1 (multi-system involvement - inflammation-dominant subtype, n = 594) is characterized by nearly universal musculoskeletal involvement (100 %) and relatively low involvement in other organ systems (cardiovascular 12 %, lungs 11.8 %, digestive system 6.6 %). In contrast, Subtype 2 (glandular-limited - high immunoglobulin subtype, n = 493) shows a more diversified pattern, with predominant glandular involvement (40.7 %) and notably higher involvement in the cardiovascular (24.7 %), pulmonary (13.4 %), and nervous systems (2.8 %) (Supplementary Table 2). Supplementary Table 1 further compares immunological markers between the two subtypes, revealing that Subtype 1 (multi-system involvement - inflammation-dominant subtype) has significantly higher levels of ESR (52.03 ± 31.69 vs. 42.16 ± 30.44 mm/h, P = 0.001), CRP (16.16 ± 25.20 vs. 12.32 ± 25.96 mg/L, P = 0.029), and RF (246.41 ± 1177.49 vs. 32.75 ± 126.74 IU/mL, P = 0.001) compared to Subtype 2 indicating stronger systemic inflammation in the multi-system inflammation-dominant subtype. Subtype 2, on the other hand, exhibits higher levels of IgG, aligning with its glandular-limited, high immunoglobulin characteristics. Supplementary Fig. S2 shows the clustering heatmap, clearly depicting the different organ involvement patterns between the two subtypes, further supporting our subtype classification.

#### 3.2.2 Comparison of clinical classification and data-driven clustering

**Fig. 4:**
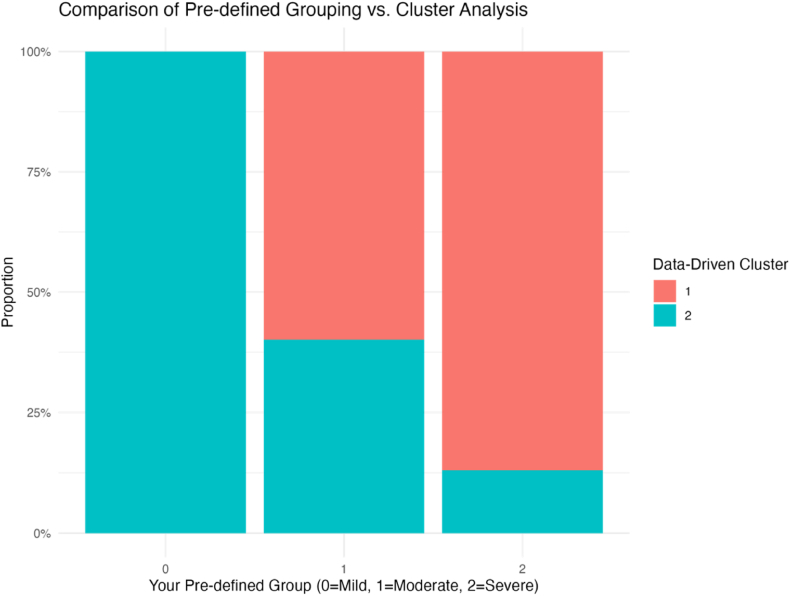
Comparison of Traditional Clinical Classification and Data-Driven Clustering. Note: The x-axis represents the traditional clinical classification (0 = mild, 1 = moderate, 2 = severe); the y-axis represents the proportion of Subtype 1 and Subtype 2 within each group. The red portion represents Subtype 1 (multi-system involvement - inflammation-dominant subtype), while the blue portion represents Subtype 2 (glandular-limited - high immunoglobulin subtype).

Fig. 4 compares the traditional clinical severity classification (mild/moderate/severe) with the data-driven clustering approach. There is substantial but not complete concordance between these classification methods. Notably, in the mild group (Group 0), nearly all patients (98.5 %) belong to Subtype 2 (glandular-limited - high immunoglobulin subtype), indicating that this mild phenotype aligns with the glandular-limited high immunoglobulin subtype identified by clustering. The moderate severity group (Group 1) shows a mixed distribution, with approximately 60.4 % belonging to Subtype 1 and 39.6 % to Subtype 2. In contrast, the severe group (Group 2) is predominantly classified as Subtype 1 (multi-system involvement - inflammation-dominant subtype, 87.3 %), suggesting that the multi-system involvement - inflammation-dominant subtype is likely associated with more severe disease manifestations as defined by traditional clinical classification.

#### 3.2.3 Independent predictors of disease subtypes

Table 2 presents the results of univariate and multivariate regression analyses, identifying independent predictors for Sjögren’s syndrome subtype classification. The multivariate model identified several factors significantly associated with being classified as Subtype 1 (multi-system involvement - inflammation-dominant subtype). Demographic factors include younger age (OR 0.978, 95 % CI 0.964–0.993, P = 0.005) and male gender (OR 2.559, 95 % CI 1.109–6.090, P = 0.005). Laboratory parameters independently associated with subtype classification include lower levels of γ-glutamyl transferase (GGT) (OR 0.993, 95 % CI 0.988–0.999, P = 0.012), lower albumin (OR 0.935, 95 % CI 0.891–0.983, P = 0.008), higher potassium levels (OR 1.607, 95 % CI 1.061–2.433, P = 0.025), and lower blood glucose (OR 0.800, 95 % CI 0.670–0.955, P = 0.013). Inflammatory markers are strong independent predictors of disease subtype classification, including elevated ESR (OR 1.007, 95 % CI 1.000–1.013, P = 0.044), higher ASO levels (OR 1.003, 95 % CI 1.000–1.005, P = 0.026), and particularly elevated rheumatoid factor (RF) (OR 1.004, 95 % CI 1.002–1.005, P = 0.001).

**Table 2:** Univariate and multivariate regression analysis of independent predictors for Sjögren’s syndrome subtype classification.

Characteristic	Univariable		Multivariable	
	OR(95 %CI)	P	OR(95 %CI)	P
Age (years)	0.987(0.978–0.997)	0.013	**0.978(0.964∼0.993)**	**0.005**
Female, n (%)	0.985(0.605–1.601)	0.950	**2.559(1.109∼6.090)**	**0.005**
BMI group, n (%)	1.170(1.017–1.347)	0.029	1.096(0.919–1.309)	0.308
Alanine Aminotransferase (U/L)	0.998(0.995–1.001)	0.185	–	
Aspartate Aminotransferase (U/L)	0.998(0.994–1.001)	0.116	–	
Alkaline Phosphatase (U/L)	0.998(0.996–1.001)	0.264	**-**	
Gamma-Glutamyl Transferase (U/L)	0.997(0.995–1.000)	0.019	**0.993(0.988∼0.999)**	**0.012**
Total Protein (g/L)	0.990(0.976–1.004)	0.145	–	
Albumin (g/L)	0.932(0.907–0.957)	0.001	**0.935(0.891∼0.983)**	**0.008**
Creatinine (μmol/L)	0.996(0.991–1.001)	0.102	–	
Cholesterol (mmol/L)	0.956(0.858–1.065)	0.414	–	
Triglyceride (mmol/L)	0.921(0.792–1.070)	0.282	–	
High-Density Lipoprotein (mmol/L)	0.681(0.478–0.970)	0.033	0.787(0.470–1.315)	0.360
Low-Density Lipoprotein (mmol/L)	1.007(0.874–1.161)	0.920	–	
Lactate Dehydrogenase (U/L)	0.999(0.998–1.001)	0.384	–	
Hydroxybutyrate Dehydrogenase (U/L)	0.999(0.997–1.001)	0.415	–	
Potassium (mmol/L)	1.521(1.134–2.040)	0.005	**1.607(1.061∼2.433)**	**0.025**
Sodium (mmol/L)	1.008(0.966–1.051)	0.725	–	
Chloride (mmol/L)	1.023(0.988–1.059)	0.204	–	
Calcium (mmol/L)	0.232(0.088–0.613)	0.003	1.786(0.337–9.464)	0.495
Magnesium (mmol/L)	1.253(0.323–4.863)	0.744	–	
Phosphate (mg/dL)	0.998(0.996–1.001)	0.277	–	
Blood Glucose (mmol/L)	0.854(0.771–0.946)	0.002	**0.800(0.670∼0.955)**	**0.013**
Globulin (g/L)	0.987(0.976–0.998)	0.023	0.998(0.982–1.013)	0.765
Immunoglobulin G (g/L)	0.995(0.983–1.008)	0.452	–	
Immunoglobulin A (g/L)	1.018(0.908–1.142)	0.757	–	
Immunoglobulin M (g/L)	1.095(0.911–1.316)	0.332	–	
Complement C3 (g/L)	1.078(0.774–1.499)	0.658	–	
Complement C4 (g/L)	2.060(0.601–7.064)	0.250	–	
SSA positive, n (%)	0.819(0.640–1.048)	0.112	–	
SSB positive, n (%)	0.947(0.700–1.280)	0.723	–	
Erythrocyte Sedimentation Rate (mm/hr)	1.010(1.006–1.015)	0.001	**1.007(1.000∼1.013)**	**0.044**
Anti-Streptolysin O (IU/mL)	1.002(1.000–1.004)	0.018	**1.003(1.000∼1.005)**	**0.026**
C-Reactive Protein (mg/L)	1.006(1.001–1.012)	0.032	0.996(0.988–1.004)	0.353
Rheumatoid Factor (IU/mL)	1.006(1.004–1.008)	0.001	**1.004(1.002∼1.005)**	**0.001**
Antihypertensive drugs, n (%)	1.008(0.759–1.339)	0.995	–	
Hypoglycemic agents, n (%)	0.996(0.578–1.717)	0.988	–	
Total Glucosides of Paeony, n (%)	0.785(0.426–1.444)	0.436	–	
Tripterygium Wilfordii n (%)	1.268(0.807–1.991)	0.303	–	
Hydroxychloroquine, n (%)	1.106(0.868–1.409)	0.414	–	
Hormones/Steroids, n (%)	1.047(0.824–1.329)	0.708	–	

Note: OR = Odds Ratio; CI = Confidence Interval; “-” indicates the variable was not included in the multivariate model; variables with statistical significance in the multivariate model are displayed in bold; Subtype 1 (multi-system involvement - inflammation-dominant subtype) = 1, Subtype 2 (glandular-limited - high immunoglobulin subtype) = 0.

#### 3.2.4 Development of a composite clinical prediction score

**Fig. 5:**
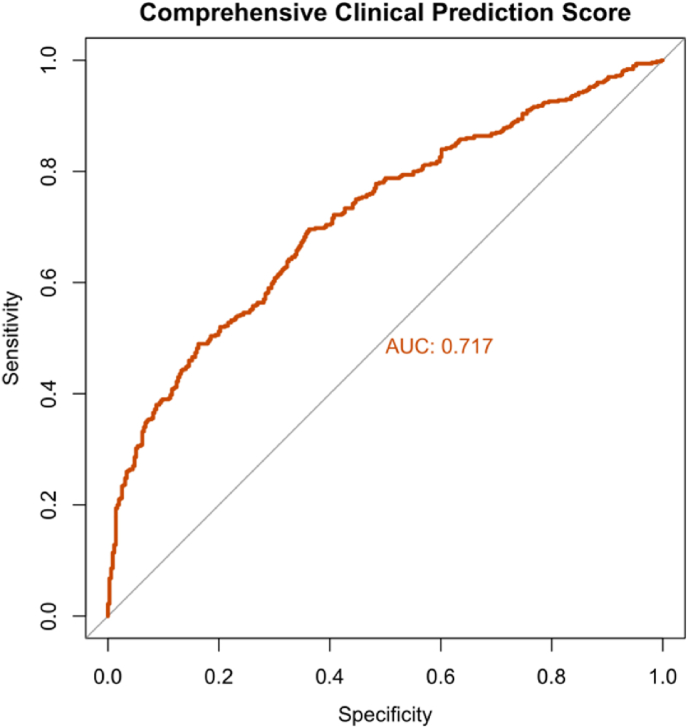
ROC curve for the composite clinical prediction score. The model achieved an AUC of 0.717 (95 % CI: 0.684–0.751). At the optimal cut-off of 0.526, sensitivity was 69.6 % and specificity was 63.8 %.

To enhance the clinical applicability of our classification system, we developed a composite prediction model using routine laboratory biomarkers identified as independent predictors in the multivariate analysis. The model incorporated RF, ESR, Albumin, Gender, Potassium (K), Glucose, GGT, and IgG.This composite score demonstrated acceptable discriminatory power with an AUC of 0.717 (95 % CI: 0.684–0.751) (Fig. 5). Using the optimal cut-off value of 0.526 determined by Youden’s index, the model achieved a sensitivity of 69.6 % and a specificity of 63.8 %.The prediction probability (P) for the Multi-System Inflammatory Phenotype (Cluster 1) can be calculated using the following formula:Logit(P) = 0.499 + (0.005 × RF) + (0.004 × ESR) − (0.047 × Albumin) + (0.180 × Male) + (0.463 × K) − (0.111 × Glucose) − (0.003 × GGT) − (0.011 × IgG). Note: Male = 1, Female = 0. Variables represent serum concentrations in standard units. The directionality of model coefficients aligns with the biological characteristics of the identified phenotypes: positive coefficients for inflammatory markers (RF, ESR) and potassium, combined with negative coefficients for albumin, glucose, and IgG, reflect the distinct metabolic and immunological profiles of each phenotype. This consistency validates the biological relevance of our data-driven clustering results and provides a practical bridge between routine laboratory metrics and complex clinical phenotypes.

#### 3.2.5 Nonlinear relationship between laboratory parameters and Sjögren’s syndrome subtype risk

**Fig. 6:**
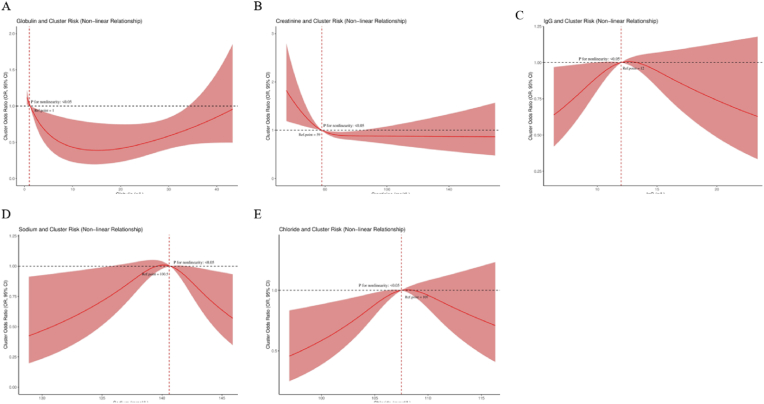
Nonlinear Relationship Between Key Laboratory Parameters and Subtype 1 (Multi-System Involvement - Inflammation-Dominant Subtype) Classification Risk. Figure Legend: Panels A–E display the nonlinear relationship between globulin, creatinine, IgG, sodium, and chloride levels and the risk of Subtype 1 (multi-system involvement - inflammation-dominant subtype) classification. The red line represents the fitted curve, and the pink shaded area indicates the 95 % confidence interval. The horizontal dashed line represents the reference line where the relative risk is 1, and the vertical dashed line indicates the inflection point where risk changes. All nonlinear relationships are statistically significant (P < 0.05). Globulin levels show a U-shaped relationship with clustering risk (P < 0.05), while low creatinine levels are associated with high risk (P < 0.05). IgG levels display an inverted U-shaped relationship with risk (P < 0.05), and both sodium and chloride levels exhibit similar inverted U-shaped relationships with risk (P < 0.05). These nonlinear relationships highlight the complex impact of laboratory parameters on subtype classification and emphasize the importance of considering the optimal range of biomarkers rather than simply categorizing them as high or low.

To further investigate the complex relationship between laboratory parameters and Sjögren’s syndrome subtype classification, we performed a nonlinear risk analysis. Fig. 6 illustrates the nonlinear relationship between five key laboratory parameters and the risk of Subtype 1 (multi-system involvement - inflammation-dominant subtype) classification. Fig. 6A shows a U-shaped relationship between globulin levels and clustering risk, with the lowest risk at moderate levels and an increased risk at both high and low extremes (P < 0.05). Fig. 6B demonstrates that creatinine levels, when below 50 mg/dL, are associated with a higher clustering risk, after which the risk stabilizes (P < 0.05). Fig. 6C depicts an inverted U-shaped relationship between IgG levels and clustering risk, with the highest risk at approximately 12 g/L, followed by a decrease in risk as the concentration increases (P < 0.05). Both serum sodium (Fig. 6D) and chloride levels (Fig. 6E) show similar inverted U-shaped relationships, peaking at approximately 138 mmol/L and 105 mmol/L, respectively (P < 0.05). These nonlinear relationships indicate that certain laboratory parameters are more complex in their association with Sjögren’s syndrome subtypes than linear models can capture, suggesting that clinical evaluations should consider the optimal range of these parameters rather than relying on simple high or low thresholds.

## 4 Discussion

This study applied unsupervised clustering and network analysis to 1087 patients with primary Sjögren’s syndrome, revealing disease phenotypes with significantly different clinical and immunological features, offering a new perspective for understanding the heterogeneity of Sjögren’s syndrome. By integrating various data-driven methods, we not only identified two distinct clinical subtypes but also explored the complex relationship between biomarkers and disease phenotypes, including nonlinear risk patterns. This multi-layered analysis represents an important step toward precision medicine strategies.

The two primary clinical subtypes identified through unsupervised clustering—multisystem inflammatory subtype (Subtype 1) and glandular-limited high immunoglobulin subtype (Subtype 2)—suggest that Sjögren’s syndrome may represent a spectrum of related diseases rather than a single homogeneous entity. The multisystem inflammatory subtype is characterized by widespread musculoskeletal involvement and significantly elevated inflammatory markers, which is highly consistent with the “arthritic” subgroup observed in previous studies [10]. Notably, this subtype is significantly associated with elevated rheumatoid factor (RF) levels, suggesting a potential pathological overlap with rheumatoid arthritis or the presence of a specific immune mechanism driving joint involvement in Sjögren’s syndrome [11]. In contrast, the glandular-limited high immunoglobulin subtype exhibits more “classic” features of Sjögren’s syndrome, with prominent glandular, cardiovascular, and pulmonary involvement, along with elevated IgG levels. This finding aligns with Koh et al.’s observation of an association between hypergammaglobulinemia and multiorgan damage [12]. However, it is important to consider that hypergammaglobulinemia is often associated with anti-Ro/SSA positivity, which may confound the relationship. Therefore, the hypothesis of “hypergammaglobulinemia driving damage” may be confounded by the antibody status, and future studies are needed to clarify the independent roles of these serological markers in the pathophysiological process of the disease. This subtype differentiation likely reflects distinct pathogenic mechanisms and immune response patterns, which could have significant implications for guiding personalized treatment.

The strong association between RF and musculoskeletal involvement revealed through network analysis is one of the key findings of this study, reinforcing the importance of RF as a biomarker for joint manifestations in Sjögren’s syndrome. This finding is consistent with the study by Brown et al. [13], which reported that RF positivity is associated with more severe joint disease in Sjögren’s syndrome patients. Importantly, our data show that RF levels are not only a strong independent predictor of subtype classification but may also be a key mediator connecting musculoskeletal manifestations with overall disease severity. This suggests that RF may not only serve as a diagnostic biomarker but could also play a role in the pathophysiological process of the disease, which aligns with recent evidence on the direct involvement of autoantibodies in the pathogenesis of autoimmune diseases [14].

The comparison between traditional clinical classification and data-driven clustering provided interesting insights. While there is substantial concordance between these methods, the mixed distribution in the moderate group suggests that traditional severity-based classification may not fully capture subtype-specific pathophysiological processes. Notably, the finding that the majority of patients in the severe group belong to the multi-system inflammatory subtype challenges the traditional view that disease severity is primarily determined by the number of affected organs. Instead, our results suggest that specific involvement patterns, particularly musculoskeletal manifestations with elevated RF, may be key drivers of perceived disease severity. This finding supports the view proposed by Fang et al. [15], that patient stratification based on clinical phenotypes may be superior to simple organ-counting methods.

The predictors of disease subtypes identified through multivariate regression analysis provide important insights into potential pathophysiological mechanisms. The association of younger age with the multi-system inflammatory subtype contrasts with some previous studies [16], which reported that older patients exhibit more systemic involvement. This discrepancy may reflect a unique disease manifestation pattern in younger patients in our cohort or suggest that age may influence immune responses through complex mechanisms. The association of male gender with the multi-system inflammatory subtype is consistent with previous research, which found that male pSS patients tend to exhibit more severe systemic disease [17]. The strong independent association between RF and disease subtypes further supports its potential role as a key biomarker in Sjögren’s syndrome, which aligns with the studies by Oskam et al. [18] and Maślińska et al. [19].

The nonlinear relationship between laboratory parameters and subtype risk revealed complex biological patterns that traditional linear models fail to capture. The U-shaped relationship between globulin levels and disease risk suggests that immune system imbalance can lead to similar clinical manifestations through various mechanisms, while the inverted U-shaped relationship with IgG levels likely reflects dynamic changes in immune response at different stages. These findings not only deepen our understanding of disease mechanisms but also suggest that clinical practice should consider the optimal range of biomarkers rather than simple high/low thresholds. Recognizing these nonlinear relationships may be crucial for developing more precise predictive models and individualized treatment strategies.

Our study has several important strengths. First, the large sample size enhances the statistical power and robustness of the findings. Second, the comprehensive application of various data-driven methods provides a multidimensional perspective on disease heterogeneity. Third, network analysis revealed complex associations that may have been overlooked by traditional statistical methods. However, there are also some limitations in our study. As a cross-sectional design, we were unable to assess the stability of subtypes over time or the patterns of disease progression, which will need to be verified through prospective longitudinal studies. Additionally, this design precludes assessment of temporal variations in biomarkers, which may fluctuate due to disease activity or treatment effects. Regarding sex differences, although male gender was identified as an independent predictor, the small proportion of male patients (6.4 %) limits comprehensive sex-stratified analyses. Furthermore, the single-center nature of the study may limit the generalizability of the results, especially considering that Sjögren’s syndrome may present differently across different ethnic groups and geographical regions. Finally, although our clustering method identified stable subtypes, the K-means algorithm itself has certain limitations, including sensitivity to the initial centroids and the assumption that clusters are spherical in shape. Moreover, due to the retrospective design of the study, we were unable to systematically collect patient-reported outcomes (PROs) such as fatigue, pain, and dryness, which makes it difficult to directly compare our identified subtypes with symptom-based clusters in the existing literature. Future prospective studies should integrate these key dimensions to gain a more comprehensive understanding of pSS phenotypes.

Based on the findings of this study, we propose several future research directions. First, validating these subtypes in independent and diverse cohorts is crucial for establishing their clinical applicability. Second, prospective longitudinal studies should explore the stability, disease progression patterns, and long-term prognosis of these subtypes. Third, molecular and genetic studies of the identified subtypes may provide deeper insights into the underlying pathophysiological mechanisms. Finally, evaluating the value of these subtypes in predicting treatment response could lay the foundation for personalized treatment strategies.

In conclusion, our study suggests that primary Sjögren’s syndrome consists of distinct clinical subtypes with different organ involvement patterns and immunological features. The data-driven classification system complements traditional clinical classifications and offers new perspectives on disease heterogeneity, potentially laying the groundwork for more individualized patient management strategies. Rheumatoid factor, as a key biomarker associated with musculoskeletal manifestations and overall disease severity, stands out and may become an important marker for future precision subtyping and targeted therapy. By integrating network analysis, clustering algorithms, and nonlinear risk assessment, this study provides a novel perspective on the complex pathophysiology of Sjögren’s syndrome and lays the foundation for future precision medicine approaches. We must emphasize, however, that given the limited effective therapies for pSS, the direct clinical value of the subtypes identified in this study for guiding current individualized treatment remains exploratory. Nonetheless, identifying these subgroups with distinct biological characteristics is a critical first step in developing and testing new targeted treatment strategies in the future.

## CRediT authorship contribution statement

**Jianbin Li:** Writing – review & editing, Writing – original draft, Visualization, Validation, Resources, Methodology, Investigation, Data curation, Conceptualization. **Suiran Li:** Writing – review & editing, Writing – original draft, Formal analysis, Data curation, Conceptualization. **Wei Liu:** Writing – review & editing, Writing – original draft, Supervision, Software, Resources, Project administration, Methodology, Investigation, Conceptualization.

## Ethics statement

This study was approved by the Ethics Committee of the First Affiliated Hospital of Tianjin University of Traditional Chinese Medicine (approval number: TYLL2018[K]026). As this study was a retrospective analysis, the Ethics Committee waived the requirement for informed consent from patients.

## Clinical trial number

Clinical trial number: not applicable.

## Consent to publish declaration

Not applicable.

## Funding

This work was supported by the following grants:

1. Project of Clinical Collaboration of Traditional Chinese Medicine and Western Medicine for Major Refractory Diseases—01 Direct Funds—Inheritance and Development of Traditional Chinese Medicine in 2024 (Batch II) (Grant No. 20240905).
2. Research on TCM Syndrome Patterns and Formulation of Expert Consensus for Rheumatic Diseases (RA, SS, Gout) (Grant No. 20240204011).
3. Research on Integrated Traditional Chinese and Western Medicine Diagnosis and Treatment Regimens for Major Refractory Diseases (Grant No. 2023382).
4. National Famous Traditional Chinese Medicine Experts Inheritance Studio (Grant No. 975022-2024).
5. Efficacy and Mechanism of Yin-Nourishing and Detoxifying Traditional Chinese Medicine in the Treatment of Sjögren's Syndrome (Grant No. GZY-KJS-2024-05).

## Declaration of competing interest

The authors declare that they have no known competing financial interests or personal relationships that could have appeared to influence the work reported in this paper.

## Data Availability

Data will be made available on request.

## References

[1] Luo D., Li L., Yang Y. (2023 Aug 10). Unraveling the transcriptome-based network of Tfh cells in primary Sjögren syndrome: insights from a systems biology approach. Front. Immunol..

[2] Fu J., Peng W., Wu Q. (2025 Jun 6). Serum growth differentiation factor 15 associates with extra-glandular manifestations and disease activity of primary Sjögren’s syndrome. Sci. Rep..

[3] Thorlacius G.E., Hultin-Rosenberg L., Sandling J.K. (2021 Feb 1). Genetic and clinical basis for two distinct subtypes of primary Sjögren’s syndrome. Rheumatology.

[4] Lan J., Deng C., Huang H. (2024 Apr 16). Seronegative primary Sjögren’s syndrome, a distinct subtype of primary Sjögren’s syndrome in Chinese patients. BMC Rheumatol.

[5] Lee J.J., Park Y.J., Park M. (2021 Sep 19). Longitudinal analysis of symptom-based clustering in patients with primary Sjogren’s syndrome: a prospective cohort study with a 5-year follow-up period. J. Transl. Med..

[6] Ferreira M.B., Kobayashi M., Costa R.Q. (2023 Nov). Unsupervised clustering to differentiate rheumatoid arthritis patients based on proteomic signatures. Scand. J. Rheumatol..

[7] Shiboski C.H., Shiboski S.C., Seror R. (2017 Jan). 2016 American college of Rheumatology/European league against rheumatism classification criteria for primary Sjögren’s syndrome: a consensus and data-driven methodology involving three international patient cohorts. Arthritis Rheumatol..

[8] Seror R., Theander E., Brun J.G. (2015 May). Validation of EULAR primary Sjögren’s syndrome disease activity (ESSDAI) and patient indexes (ESSPRI). Ann. Rheum. Dis..

[9] Guo Y., Gong B., Li Y. (2025 Jun 24). Non-invasive prediction of NSCLC immunotherapy efficacy and tumor microenvironment through unsupervised machine learning-driven CT radiomic subtypes: a multi-cohort study. Int. J. Surg..

[10] Avouac J., Walker U., Tyndall A. (2010 Jul). Characteristics of joint involvement and relationships with systemic inflammation in systemic sclerosis: results from the EULAR scleroderma trial and research group (EUSTAR) database. J. Rheumatol..

[11] Charles E.D., Orloff M.I., Dustin L.B. (2011 Jan 5). A flow cytometry-based strategy to identify and express IgM from VH1-69+ clonal peripheral B cells. J. Immunol. Methods.

[12] Koh J.H., Park Y., Lee J. (2021 Nov-Dec). Hypergammaglobulinaemia predicts glandular and extra-glandular damage in primary Sjögren’s syndrome: results from the KISS cohort study. Clin. Exp. Rheumatol..

[13] Brown L.E., Frits M.L., Iannaccone C.K. (2015 May). Clinical characteristics of RA patients with secondary SS and association with joint damage. Rheumatology.

[14] Wu Y., Luo J., Duan L. (2024 Aug 30). Pathogenic mechanisms of disease in idiopathic inflammatory myopathies: autoantibodies as clues. Front. Immunol..

[15] Fang Q., Ou J., Nandakumar K.S. (2019 Oct 27). Autoantibodies as diagnostic markers and mediator of joint inflammation in arthritis. Mediat. Inflamm..

[16] Seeliger T., Kramer E., Konen F.F. (2023 Jun). Sjögren’s syndrome with and without neurological involvement. J. Neurol..

[17] Ramírez Sepúlveda J.I., Kvarnström M., Brauner S. (2017 May 12). Difference in clinical presentation between women and men in incident primary Sjögren’s syndrome. Biol. Sex Differ..

[18] Oskam N., Ooijevaar-De Heer P., Kos D. (2023 Jul). Rheumatoid factor autoantibody repertoire profiling reveals distinct binding epitopes in health and autoimmunity. Ann. Rheum. Dis..

[19] Maślińska M., Mańczak M., Kwiatkowska B. (2019 May). Usefulness of rheumatoid factor as an immunological and prognostic marker in PSS patients. Clin. Rheumatol..

