# Corrigendum to “Data-driven classification of primary Sjögren's syndrome: From cluster analysis to clinical immune phenotypes and predictive biomarkers” [J. Transl. Autoimmun. 12 (2026) 100338]

**DOI:** 10.1016/j.jtauto.2025.100343

**Published:** 2025-12-11

**Authors:** Jianbin Li, Suiran Li, Wei Liu

**Affiliations:** a Department of Rheumatism and Immunity, First Teaching Hospital of Tianjin University of Traditional Chinese Medicine, Tianjin, China; b National Clinical Research Center for Chinese Medicine Acupuncture and Moxibustion, Tianjin, China

The authors regret that due to a layout/formatting error during the production process, the data in Table 1 became misaligned, resulting in discrepancies between the table and the corresponding text description in the Results section (Section 3.1).

**The corrected Table 1 and text are provided below:**

**Corrected Section 3.1 Text:**

Table 1 presents the demographic and clinical characteristics of 1087 patients with Sjögren's syndrome, stratified by disease severity (mild, moderate, and severe). The mean age of all patients was 58.25 ± 12.16 years, with a predominance of females (93.56 %). No significant differences were observed in age, gender distribution, or BMI across the severity groups.

Several laboratory parameters showed significant differences between severity groups. Liver function indicators, particularly ALT, were significantly lower in the moderate and severe groups compared to the mild group (P = 0.024). Total protein and albumin levels decreased with increasing disease severity (P = 0.029 and P = 0.001, respectively). Inflammatory markers were significantly associated with disease severity, with ESR and CRP levels gradually increasing from mild to severe groups (P = 0.003 and P = 0.015, respectively). Notably, rheumatoid factor (RF) significantly increased with disease severity, rising from 25.82 ± 52.74 IU/mL in the mild group to 275.36 ± 1707.09 IU/mL in the severe group (P = 0.041).

Major organ involvement patterns differed significantly between severity groups. The mild group was primarily characterized by glandular involvement, with minimal non-glandular manifestations. The moderate group showed increased involvement of the musculoskeletal system (393 cases), cardiovascular system (111 cases), and lungs (65 cases). The severe group exhibited the highest rates of involvement in the musculoskeletal, lungs, cardiovascular, digestive, and endocrine systems (all P < 0.05).

Regarding comorbidities, hypertension was the most common (171 cases), followed by pulmonary interstitial fibrosis (62 cases) and diabetes (33 cases). These comorbidities exhibited significant differences between severity groups (P < 0.01), with higher prevalence rates in the moderate and severe groups. Notably, lymphoma, though rare (7 cases), had a significantly higher prevalence in the severe group (P = 0.001).

**Corrected Table 1: Demographic Characteristics and Clinical Manifestations of Sjögren's Syndrome Patients**

**Table:** 

Character	Total (n = 1087)	Mild Group (n = 200)	Moderate Group (n = 656)	Severe Group (n = 231)	P-value
Age (years)	58.25 ± 12.16	56.88 ± 12.152	58.61 ± 12.269	58.41 ± 11.806	0.205
Female, n (%)	1017 (93.56)	188 (94.00)	612 (93.29)	217 (93.94)	0.906
BMI (kg/m^2^)	23.77 ± 4.76	23.62 ± 4.27	23.73 ± 4.73	24.05 ± 5.29	0.661
Alanine Aminotransferase (U/L)	25.19 ± 40.09	31.51 ± 63.35	24.76 ± 34.26	20.99 ± 26.97	0.024
Aspartate Aminotransferase (U/L)	28.36 ± 58.99	34.26 ± 76.91	28.85 ± 62.43	21.94 ± 12.90	0.095
Alkaline Phosphatase (U/L)	79.05 ± 43.43	78.51 ± 50.53	79.44 ± 43.18	78.41 ± 37.32	0.936
Gamma-Glutamyl Transferase (U/L)	36.21 ± 65.23	37.19 ± 70.85	36.98 ± 64.94	33.20 ± 61.09	0.734
Total Protein (g/L)	68.19 ± 8.52	69.09 ± 8.94	68.34 ± 8.69	66.97 ± 7.51	0.029
Albumin (g/L)	35.79 ± 4.65	36.87 ± 4.41	35.64 ± 4.74	35.29 ± 4.46	0.001
Creatinine (μmol/L)	61.97 ± 25.13	61.54 ± 19.29	62.82 ± 28.28	59.91 ± 19.49	0.316
Cholesterol (mmol/L)	4.39 ± 1.13	4.46 ± 1.23	4.37 ± 1.10	4.38 ± 1.14	0.675
Triglyceride (mmol/L)	1.35 ± 0.81	1.39 ± 0.91	1.35 ± 0.80	1.34 ± 0.77	0.816
High-Density Lipoprotein (mmol/L)	1.12 ± 0.34	1.17 ± 0.37	1.12 ± 0.34	1.10 ± 0.34	0.125
Low-Density Lipoprotein (mmol/L)	2.62 ± 0.86	2.62 ± 0.91	2.62 ± 0.86	2.63 ± 0.83	0.98
Lactate Dehydrogenase (U/L)	190.56 ± 85.10	188.93 ± 74.45	192.20 ± 94.21	187.34 ± 64.11	0.735
Hydroxybutyrate Dehydrogenase (U/L)	143.85 ± 66.04	142.53 ± 55.45	145.53 ± 73.55	140.26 ± 50.15	0.568
Potassium (mmol/L)	3.87 ± 0.42	3.83 ± 0.45	3.87 ± 0.42	3.89 ± 0.39	0.32
Sodium (mmol/L)	140.32 ± 2.87	140.32 ± 2.74	140.34 ± 2.97	140.30 ± 2.71	0.983
Chloride (mmol/L)	107.46 ± 3.51	107.64 ± 3.70	107.43 ± 3.58	107.41 ± 3.13	0.742
Calcium (mmol/L)	2.18 ± 0.12	2.19 ± 0.13	2.18 ± 0.13	2.17 ± 0.12	0.19
Magnesium (mmol/L)	0.85 ± 0.09	0.86 ± 0.09	0.86 ± 0.09	0.85 ± 0.09	0.207
Phosphate (mg/dL)	78.93 ± 43.55	78.12 ± 50.83	79.44 ± 43.18	78.21 ± 37.61	0.897
Blood Glucose (mmol/L)	5.04 ± 1.34	4.90 ± 0.97	5.10 ± 1.47	4.99 ± 1.23	0.156
Globulin (g/L)	5.15 ± 10.53	6.85 ± 12.10	4.89 ± 10.33	4.42 ± 9.50	0.037
Immunoglobulin G (g/L)	12.69 ± 18.55	12.34 ± 3.92	12.93 ± 23.69	12.35 ± 3.54	0.878
Immunoglobulin A (g/L)	2.39 ± 1.04	2.38 ± 1.12	2.35 ± 0.99	2.52 ± 1.10	0.112
Immunoglobulin M (g/L)	1.26 ± 0.65	1.28 ± 0.67	1.23 ± 0.59	1.35 ± 0.80	0.07
Complement C3 (g/L)	1.09 ± 0.36	1.07 ± 0.36	1.09 ± 0.36	1.10 ± 0.34	0.579
Complement C4 (g/L)	0.22 ± 0.10	0.22 ± 0.09	0.22 ± 0.11	0.23 ± 0.09	0.95
Erythrocyte Sedimentation Rate (mm/hr)	47.90 ± 31.53	40.44 ± 30.24	49.29 ± 31.94	50.36 ± 30.69	0.003
Anti-Streptolysin O (IU/mL)	89.89 ± 79.67	88.96 ± 55.11	88.07 ± 81.34	95.99 ± 91.98	0.506
C-Reactive Protein (mg/L)	14.56 ± 25.57	9.56 ± 18.17	15.10 ± 25.96	17.33 ± 29.22	0.015
Rheumatoid Factor (IU/mL)	157.40 ± 908.86	25.82 ± 52.74	156.12 ± 590.06	275.36 ± 1707.09	0.041
SSA positive, n (%)	671 (61.73 %)	126 (63.00 %)	402 (61.28 %)	143 (61.90 %)	0.907
SSB positive, n (%)	211 (19.41 %)	39 (19.50 %)	126 (19.21 %)	46 (19.91 %)	0.973
No Extra-glandular Involvement, n (%)	202 (18.58 %)	200 (100.00 %)	2 (0.30 %)	0 (0.00 %)	0.001
Pulmonary system involvement, n (%)	136 (12.51 %)	0 (0.00 %)	65 (9.91 %)	71 (30.74 %)	0.001
Nervous system involvement, n (%)	14 (1.29 %)	0 (0.00 %)	12 (1.83 %)	2 (0.87 %)	0.108
Hematologic system involvement, n (%)	22 (2.02 %)	0 (0.00 %)	10 (1.52 %)	12 (5.19 %)	0.001
Hepatic system involvement, n (%)	7 (0.64 %)	0 (0.00 %)	5 (0.76 %)	2 (0.87 %)	0.445
Renal system involvement, n (%)	1 (0.09 %)	0 (0.00 %)	1 (0.15 %)	0 (0.00 %)	0.720
Musculoskeletal system involvement, n (%)	594 (54.65 %)	0 (0.00 %)	393 (59.91 %)	201 (87.01 %)	0.001
Cardiovascular system involvement, n (%)	193 (17.76 %)	0 (0.00 %)	111 (16.92 %)	82 (35.50 %)	0.001
Digestive system involvement, n (%)	83 (7.64 %)	0 (0.00 %)	29 (4.42 %)	54 (23.38 %)	0.001
Endocrine system involvement, n (%)	52 (4.78 %)	0 (0.00 %)	30 (4.57 %)	22 (9.52 %)	0.001
Hypertension, n (%)	171 (15.73 %)	0 (0.00 %)	99 (15.09 %)	72 (31.17 %)	0.001
Diabetes, n (%)	33 (3.04 %)	0 (0.00 %)	21 (3.20 %)	12 (5.19 %)	0.007
Hashimoto's thyroiditis, n (%)	12 (1.10 %)	0 (0.00 %)	6 (0.91 %)	6 (2.60 %)	0.028
Interstitial fibrosis of lung, n (%)	62 (5.70 %)	0 (0.00 %)	22 (3.35 %)	40 (17.32 %)	0.001
Lymphoma, n (%)	7 (0.64 %)	0 (0.00 %)	1 (0.15 %)	6 (2.60 %)	0.001
Malignant tumor, n (%)	14 (1.29 %)	0 (0.00 %)	8 (1.22 %)	6 (2.60 %)	0.056
Other autoimmune diseases, n (%)	496 (45.63 %)	0 (0.00 %)	300 (45.73 %)	196 (84.85 %)	0.001
Medication Use					
Antihypertensive drugs, n (%)	250 (23.00 %)	27 (13.50 %)	160 (24.38 %)	63 (27.24 %)	0.001
Antidiabetic Meds, n (%)	55 (5.06 %)	12 (6.00 %)	35 (5.34 %)	8 (3.46 %)	0.428
Hormones/Steroids, n (%)	211 (19.43 %)	29 (14.50 %)	124 (18.91 %)	58 (25.11 %)	0.018
Tripterygium Wilfordii, n (%)	85 (7.81 %)	15 (7.50 %)	51 (7.78 %)	19 (8.22 %)	0.959
Hydroxychloroquine, n (%)	460 (42.32 %)	81 (40.50 %)	283 (43.14 %)	96 (41.56 %)	0.776
Total Glucosides of Paeony, n (%)	43 (3.95 %)	14 (7.00 %)	24 (3.66 %)	5 (2.16 %)	0.030

Note: Data are presented as mean ± standard deviation or n (%). A P-value <0.05 was considered statistically significant. Group comparisons were performed using one-way analysis of variance (ANOVA) for continuous variables and chi-square test for categorical variables.

The authors would like to apologise for any inconvenience caused.

