# Supplementary material for: Data-driven classification of primary Sjögren’s syndrome: From cluster analysis to clinical immune phenotypes and predictive biomarkers
Source: J Transl Autoimmun. 2025 Dec 3;12:100338. doi: 10.1016/j.jtauto.2025.100338 (PMC12721314; doi:10.1016/j.jtauto.2025.100338)
Supplement: Multimedia component 1 [file mmc1.docx]

Supplementary Table 1: Comparison of Immunological Features Between the Two Sjögren’s Syndrome Subtypes

| Characteristic | Subtype 1 (n=594) | Subtype 2 (n=493) | P-value |
| --- | --- | --- | --- |
| IgG (g/L) | 12.18 ± 3.25 | 13.32 ± 27.32 | 0.314 |
| IgA (g/L) | 2.40 ± 1.01 | 2.38 ± 1.08 | 0.757 |
| IgM (g/L) | 1.28 ± 0.68 | 1.25 ± 0.63 | 0.331 |
| C3 (g/L) | 1.10 ± 0.36 | 1.09 ± 0.37 | 0.658 |
| C4 (g/L) | 0.23 ± 0.11 | 0.22 ± 0.09 | 0.248 |
| SSA positive (%)¹ | 317（64.30） | 354（59.59） | 0.112 |
| SSB positive (%)¹ | 98（19.87） | 113（19.02） | 0.723 |
| ESR (mm/h) | 52.03 ± 31.69 | 42.16 ± 30.44 | 0.001 |
| CRP (mg/L) | 16.16 ± 25.20 | 12.32 ± 25.96 | 0.029 |
| RF (IU/mL) | 246.41 ± 1177.49 | 32.75 ± 126.74 | 0.001 |

Supplementary Table 2: Comparison of Organ Involvement Between the Two Sjögren’s Syndrome Subtypes

| System Involvement | Subtype 1 (n=594) | Subtype 2 (n=493) | P-value |
| --- | --- | --- | --- |
| Glandular involvement, n (%) | 1 (0.17%) | 201 (40.7%) | 0.001 |
| Pulmonary system involvement, n (%) | 70 (11.8%) | 66 (13.4%) | 0.426 |
| Nervous system involvement, n (%) | 0 (0%) | 14 (2.8%) | 0.001 |
| Hematologic system involvement, n (%) | 6 (1.0%) | 16 (3.2%) | 0.009 |
| Hepatic system involvement, n (%) | 2 (0.34%) | 5 (1.0%) | 0.164 |
| Renal system involvement, n (%) | 0 (0%) | 1 (0.2%) | 0.272 |
| Musculoskeletal system involvement, n (%) | 594 (100%) | 0 (0%) | 0.001 |
| Cardiovascular system involvement, n (%) | 71 (12.0%) | 122 (24.7%) | 0.001 |
| Digestive system involvement, n (%) | 39 (6.6%) | 44 (8.9%) | 0.145 |
| Endocrine system involvement, n (%) | 22 (3.7%) | 30 (6.1%) | 0.067 |


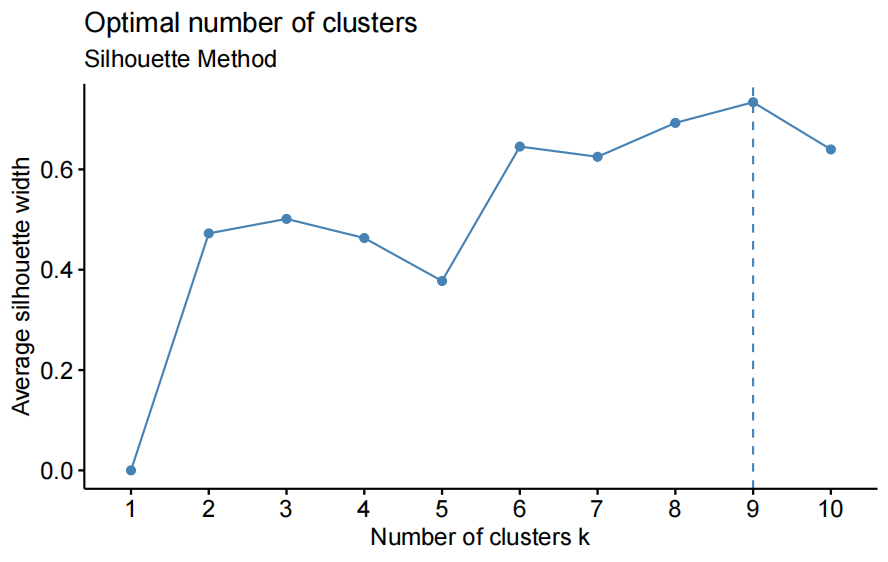


Supplementary Figure S1: Silhouette Coefficient Plot for Determining the Optimal Number of Clusters

Figure Legend: The x-axis represents the number of clusters (k), and the y-axis represents the average silhouette coefficient value. Higher silhouette coefficient values indicate better clustering quality. The silhouette coefficient reaches a reasonably high value at k=2, which is selected as the optimal number of clusters.


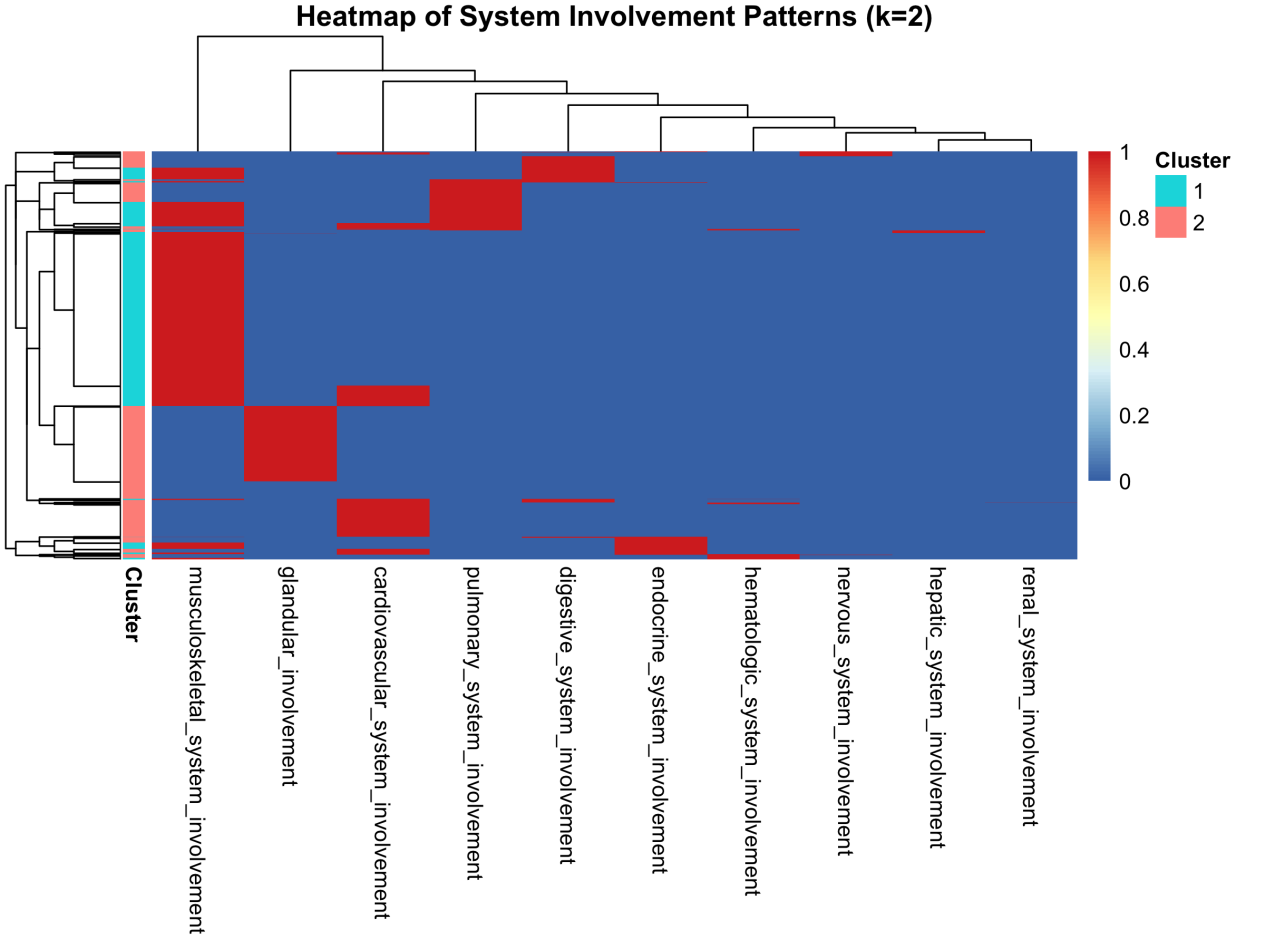


Supplementary Figure S2: Clustering Heatmap of Organ Involvement Patterns in Sjögren’s Syndrome

Figure Legend: Rows represent patients, and columns represent different organ system involvements. Red indicates the presence of involvement (1), and blue indicates the absence of involvement (0). The colored bar on the left indicates the subtype of each patient (blue = Subtype 1, pink = Subtype 2). This heatmap visually demonstrates the distinct organ involvement patterns in each subtype, supporting the identification of two clinically relevant subtypes.
